# Joint-Preserving Surgery in Varus Ankle Osteoarthritis

**DOI:** 10.3390/jcm11082194

**Published:** 2022-04-14

**Authors:** Ahmad Alajlan, Simone Santini, Faisal Alsayel, Kar H. Teoh, Waheeb Alharbi, Luise Puls, Carlo Camathias, Mario Herrera-Pérez, Sergio Tejero, Alexej Barg, Martin Wiewiorski, Victor Valderrabano

**Affiliations:** 1 Department of Orthopedic Surgery, Security Forces Hospital, P.O. Box 3643, Riyadh 11481, Saudi Arabia; dr-ahmad-a@hotmail.com; 2 Department of Orthopaedics and Trauma Surgery, University Campus Bio-Medico of Rome, 00128 Rome, Italy; s.santini@unicampus.it; 3 Department of Orthopedic Surgery, King Fahad Specialist Hospital, P.O. Box 15215, Dammam 31444, Saudi Arabia; alsayel002@gmail.com; 4 Princess Alexandra Hospital NHS Trust, Harlow CM20 1QX, UK; karhao@gmail.com; 5 King Fahad Armed Forces Hospital, Al Kurnaysh Rd, Al Andalus, Jeddah 23311, Saudi Arabia; dr.waheeb.alsaadi@gmail.com; 6 Swiss Ortho Center, Swiss Medical Network, Schmerzklinik Basel, Hirschgasslein 15, 4010 Basel, Switzerland; luise.puls1@gmail.com; 7 Medical Faculty, University of Basel, 4056 Basel, Switzerland; camathias.carlo@gmail.com; 8 Head Foot and Ankle Unit, Orthopaedic Department, University of La Laguna, 38200 San Cristóbal de La Laguna, Spain; herrera42@gmail.com; 9 Foot and Ankle Unit, Orthopedic Surgery and Traumatology Service, Hospital Universitario Virgen del Rocío, Av. Manuel Siurot, s/n, 41013 Sevilla, Spain; tejerogarciasergio@gmail.com; 10 Department of Orthopaedics, University of Utah, 590 Wakara Way, Salt Lake City, UT 84108, USA; alexej.barg@googlemail.com; 11 WinOrtho, Privatklinik Lindberg, Schickstrasse 11, 8400 Winterthur, Switzerland; wiewiorskim@gmail.com

**Keywords:** supramalleolar osteotomy, ankle osteoarthritis, varus ankle, joint preserving surgery

## Abstract

Ankle deformity is a disabling condition especially if concomitant with osteoarthritis (OA). Varus ankle OA is one of the most common ankle OA deformities. This deformity usually leads to unequal load distribution in the ankle joint and decreases joint contact surface area, leading to a progressive degenerative arthritic situation. Varus ankle OA might have multiple causative factors, which might present as a single isolated factor or encompassed together in a single patient. The etiologies can be classified as post-traumatic (e.g., after fractures and lateral ligament instability), degenerative, systemic, neuromuscular, congenital, and others. Treatment options are determined by the degree of the deformity and analyzing the pathology, which range from the conservative treatments up to surgical interventions. Surgical treatment of the varus ankle OA can be classified into two categories, joint-preserving surgery (JPS) and joint-sacrificing surgery (JSS) as total ankle arthroplasty and ankle arthrodesis. JPS is a valuable treatment option in varus ankle OA, which should not be neglected since it has showed a promising result, optimizing biomechanics and improving the survivorship of the ankle joint.

## 1. Introduction

Varus ankle osteoarthritis (OA) is caused by a deviation of the ankle’s mechanical and anatomical axes toward the medial. Asymmetric cartilage attrition and joint abnormalities are caused by uneven tension on the joint surface, which eventually leads to OA [1].

The medial talar dome and tibia plafond, as well as the medial gutter of the tibiotalar joint, are commonly affected. Varus ankle OA is characterized by talus malpositioning, which includes medial talus translation, internal rotation along the longitudinal talus axis, and/or varus talar tilt [2]. The misalignment of the talus causes an eccentric load on the ankle when it is weight-bearing, which can exacerbate varus cartilage degradation [3].

The most common etiology of ankle OA is post-traumatic [4].

Several surgical techniques for various stages of ankle OA have been documented. Joint-preserving surgery (JPS) and joint-sacrificing surgery (JSS) are the two types of procedures. Osteochondral resurfacing treatments, distraction arthroplasty, ankle arthroscopic debridement, and periankle corrective osteotomies are all joint-preserving operations [5].

Graehl et al. [5] in 1987 performed a supramalleolar osteotomy on eight patients symptomatic from a malunion of the distal two thirds of the tibia leading to varus ankle.

Takakura et al. [6] performed in 1995 a low tibial osteotomy on 18 patients affected by post-traumatic ankle varus osteoarthritis, and Hintermann et al. [7] also used this type of osteotomy for malunited pronation-external rotation fractures of the ankle, both with good outcomes.

Joint-sacrificing surgeries (JSS) consist in total ankle arthroplasty (TAA) and ankle arthrodesis. They both have their own advantages and an important role in varus ankle OA treatment, but they also might have long-term issues [5]. Since patients with post-traumatic ankle OA become symptomatic around 12 to 15 years earlier than patients with knee or hip OA [8], joint preserving, and long-lasting pain-relieving surgery is mandatory. The aim of this paper is to provide a review of the varus ankle OA issues and their treatments, conservatively and surgically. A description of JPS surgical technique, preoperative planning, and postoperative protocol is also provided.

## 2. Etiology and Biomechanics of the Varus Ankle and Hindfoot

### 2.1. Etiology

Ankle joint is rarely affected by primary OA. The most prevalent etiology of ankle OA is post-traumatic, with varus being the most common malalignment, according to previous clinical and epidemiologic investigation, most commonly following ankle fractures and lower leg fractures [1,3] and/or repetitive lateral ankle sprains/lateral chronic ankle instability [9]. (Table 1). Bone abnormalities, chronic ligament insufficiency, muscular imbalance, or a combination of these factors can all contribute to a varus deformity. Neurologic illnesses can have a significant impact on the development of varus deformity. These pathologic disorders start out as correctable malformations, but they can become stiff with time, causing aberrant biomechanics in the foot and ankle, as well as subsequent complications, resulting in secondary OA with fixed varus or cavovarus deformity.

### 2.2. Biomechanics

A deformity in the lower leg, ankle, hindfoot, midfoot, or forefoot causes ankle varus and cavovarus deformities. When the problem occurs in the hindfoot, as it is in patients with lateral chronic ankle instability and peroneal brevis muscle weakening, the talus makes a pathologic internal rotation and anterior subluxation, resulting in an asymmetric medial ankle OA over time [10]. When this happens, the forefoot goes into supination and the hindfoot goes into varus. With such an internally rotated foot deformity, the patient is unable to walk. As a result, the forefoot attempts to compensate by flexing the Metatarsus primus due to an overactive Peroneus longus muscle. The patient walks with a varus hindfoot and a plantigrade forefoot in this position, creating over time also a varus ankle OA model [10].

In essence, as Apostle et al. mention in their article, most of the deformity in a varus ankle might arise from the foot’s attempt at compensation. Over time, these compensatory mechanisms in turn become additional deforming factors [10].

However, the patient is typically in a vicious circle: shifting of the mechanical axis leads to unequal load distribution and decreases the surface contact forces and compartment overload [10]. Regardless of the primary etiology, the supporters of the ankle joint, soft tissue (lateral ligament insufficiency and deltoid ligament contracture), and bony tissue (tibia, talus, and calcaneus) both are eventually elicited and subject to degenerative changes. Therefore, proper management to cut the cascade is needed to stop the progression [10].

## 3. Clinical Assessment

A varus alignment is not always limited to the ankle. It is important to expose all of the lower limb in order to estimate the axis and any other deformity. The patient is inspected barefoot, both when walking and standing [11]. The alignment, deformities, and foot/ankle/hindfoot position are visually assessed [2]. An examination of the foot in standing position may reveal heel varus, cavus, and/or first-ray plantarflexion (forefoot-driven hindfoot varus). To measure the influence of the first ray on the varus hindfoot position and flexibility, the Coleman Block test can be used [10]. Tender regions along the path of the ankle joint medial, ventral, lateral, and posterior are given extra attention during palpation, as well as lateral and medial ligament complexes [2]. In addition, all the tendons around the ankle/hindfoot should be checked for tenderness. Then, range of motion (ROM) of the tibiotalar joint (plantarflexion/dorsiflexion) and subtalar joint (eversion/inversion) is measured, as well as of the Chopart joint [12]. Following the ROM, the ligament stability of the lateral ankle ligaments and Deltoid medial is documented [2]. Muscle atrophy and functional status should be checked. Muscle force evaluation should be checked by M0-5, in all main movement planes: plantarflexion, dorsiflexion, inversion, and eversion [12]. Further, particular attention should be paid to any potential tightness, such as to the Gastocnemius–Soleus complex or the posterior tibial muscle [12]. Finally, the patient’s neurovascular state should be assessed, with particular attention paid to the integrity of the peroneal and tibial nerves.

### 3.1. Imaging Modalities

Standing X-rays are required as part of the radiographic evaluation: dorsoplantar and lateral plain radiographs of the foot, ankle AP mortise, and hindfoot alignment view (Saltzman view). Furthermore, whole-leg radiographs are required to evaluate osseous abnormalities across the lower extremity, as hip and knee influence the overall alignment. Magnetic resonance imaging (MRI) is helpful to assess the cartilage of the ankle joint, ligaments, tendon, and muscular pathologies [13]. Preoperative CT scans, such as SPECT-CT [11] or a weight-bearing CT (WBCT), can aid to better comprehend the case, evaluate the bone quality, and locate cysts.

According to the Takakura staging system, there are four grades of a varus ankle osteoarthritis on weight-bearing anteroposterior (AP) radiographs: (1) Physiologic joint-space, osteophytes and early sclerosis; (2) Narrowing of the join space, only on the medial side; (3A) Subchondral bone contact with obliteration of the medial malleolus’ joint space; (3B) Subchondral bone contact with joint space obliteration extending to the talar dome’s roof; (4) Obliteration of the entire joint surface, bone on bone contact [14].

A categorical contraindication to performing a supramalleolar osteotomy is end-stage ankle osteoarthritis involving more than half of the tibiotalar joint surface [15], so a preop MRI study is necessary to assess the precise localization and extension of the chondral damage [12].

### 3.2. Preoperative Radiological Angle Measurements

**Figure 1:**
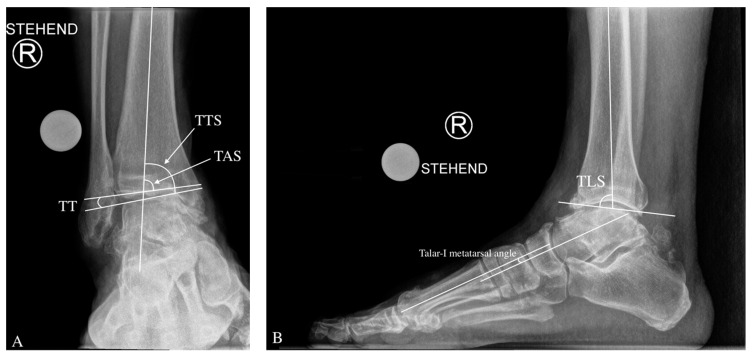

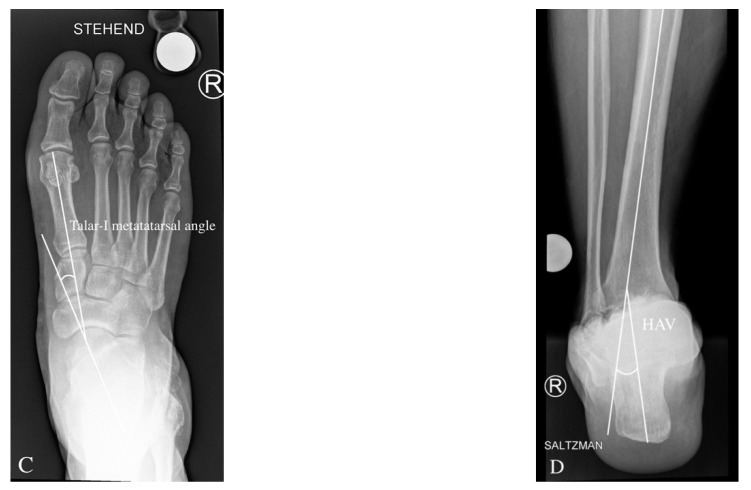
Angles for the Radiological Evaluation of a Varus Ankle Osteoarthritis. On the anteroposterior view, tibial anterior surface angle TAS (also known as medial distal tibial angle MDTA) tibiotalar surface (TTS) and talar tilt (TT) angle are shown (**A**). On lateral ankle radiograph, the tibial lateral surface angle (TLS) is assessed (**B**). The talar-1st metatarsal angle gives us a hint about the amount of midfoot deformity, both on lateral and dorsoplantar view (**B**,**C**). Inframalleolar deformity can be evaluated on the Saltzman view by measuring the hindfoot alignment view (HAV) angle (**D**).

On the anteroposterior view, the medial distal–tibial angle (MDTA: normal range 93.3 ± 3.2 degrees, also known as tibial anterior surface angle (TAS) formed by the mechanical axis of the tibia and the tangent of the tibial plafond on the medial side of the ankle (Figure 1). The MDTA in varus ankle OA is <90° and is overcorrected postoperatively over the physiological average by 2–5°.

Further, in the ap view, the tibiotalar surface angle (TTS; normal range; 87.2 ± 2.8 degrees) is measured [13,14]. The TTS in varus ankle OA is <84.4°.

The talar tilt (TT) angle is used to assess tibiotalar congruence. The tibial articular surface and the talar articular surface on the AP X-ray produce this angle. It is usually less than 4 degrees [15].

In the AP view, these criteria can be used to assess syndesmotic integrity: The tibiofibular clear space (TFCS) has a normal range of 6 mm, and the tibiofibular overlap (TFO) has a normal range of >1 mm in mortise view and >6 mm in AP view [16].

In the mortise view, disruption of Shenton’s line and the dime sign/Weber circle at the tip of the fibula/lateral talar process, frequently seen in anomalous fibular length [17,18].

On a lateral ankle radiograph, the tibial lateral surface (TLS) angle is measured using the mechanical axis of the tibia and a line passing across the ends of the tibial articular surface in a lateral view, with a typical range of 83.0 ± 3.6 degrees [19].

Inframalleolar deformity can be evaluated on the (Saltzman view) by measuring the hindfoot alignment view (HAV) angle between the anatomical axis of the tibia and the axis of the calcaneus. Normally, HAV has a value of 0–5° [20]. The Saltzman view can also be useful for detecting subtalar malalignment [21].

The lateral talar-1st metatarsal angle is an index of midfoot deformity magnitude. Pes planus is defined as a downward convex angle larger than 4° [22].

### 3.3. Conservative Treatment of Varus Ankle Osteoarthritis

There are many conservative treatments available for treating ankle OA. They all are symptomatic, with the main purpose of pain alleviation. Since the amount of specific literature for ankle OA is low, the choice is usually based on physician experience or patient tendencies. It is advised that the conservative treatment be for at least 3–6 months, before evaluating a surgical treatment [23].

Oral nonsteroidal anti-inflammatory drugs (NSAIDs) certainly have a big role in addressing pain and inflammation both. Although there are few scientific publications on the usefulness of NSAIDS in the treatment of ankle OA, their value has been demonstrated in a number of level I arthritic trials [23].

The use of viscosupplementation oral (glucosamine or chondroitin sulfate) or by injection (hyaluronic acid) has a place in the treatment of ankle OA.

The clinical benefit of oral viscosupplementation with glucosamine or chondroitin sulfate in knee OA has been demonstrated in several studies [24] However, the effect of this treatment in patients with ankle OA remains underexplored. Nevertheless, first results show that oral viscosupplementation could also be effective in ankle OA [25].

A substantial improvement in OA ankles was reported in a prospective randomized double-blind trial following 1 and 6 months of 5 weekly hyaluronic acid injections [26]. In addition, three weekly injections have been found capable to improve the Ankle Osteoarthritis Scale and American Orthopedic Foot and Ankle Society Hindfoot Score [27].

In orthopedic surgery, platelet-rich plasma (PRP) has gained popularity since it is thought to biologically stimulate musculoskeletal tissues repair, but for now its use in foot and ankle applications is supported only by limited clinical evidence [28].

The use of corticosteroid injection can decrease inflammation and pain [29], but due to its catabolic nature and the risk of damaging soft tissues, it is not for long-term use [23].

Physical therapies are frequently suggested to help increase strength and ROM. Training of peroneal muscles, proprioception exercises, Achilles tendon stretching, ankle mobilization, and gait education are examples of the main core of a physical therapy program. Muscle strength in dorsiflexion and plantarflexion has been reported to be reduced in those with ankle arthritis [30].

Since in varus ankle OA the cartilage is more compromised on the medial side, the use of insoles with lateral wedge can shift the load axis to the unaffected side, therefore unloading the osteoarthritic side of the joint [23].

### 3.4. Surgical Treatment of the Varus Ankle Osteoarthritis

Once nonoperative measures have been used without improvement, surgical intervention is indicated. Especially in patients whose symptoms increase in terms of pain, instability, or progress in radiological arthritic changes. The surgical options are joint-preserving surgery (JPS) or joint-sacrificing surgery (JSS), i.e., total ankle arthroplasty or ankle arthrodesis [5]. A painless/pain-free, plantigrade, completely functional, and stable ankle and foot is the goal of any reconstructive surgery. Recognizing the associated deformities, such as lateral chronic ankle instability, varus heel, hindfoot OA, overfiring of muscles (posterior tibial muscle and peroneus longus), insufficiency of muscles (peroneus brevis), forefoot malalignment, lesser toes deformities, Achilles tendon tightness, knee deformities, and contralateral lower limb malalignment is required for effective treatment of the varus ankle [5].

### 3.5. Joint-Preserving Surgery (JPS)

Choosing the type of surgical intervention and optimizing it depends on analyzing the pathology (etiological factors), in addition to addressing the associated comorbidity and the proper time for the surgical intervention. Table 2 summarizes the main indications and contraindications for a JPS. The key indication is varus ankle OA with a lateral partially maintained tibiotalar joint (at least 50%). A supramalleolar osteotomy (SMOT) can also be used to correct the hindfoot realignment before a total ankle arthroplasty. Patients aged >70 and hindfoot instability are a relative contraindication, while absolute contraindications are end-stage OA and hindfoot instability unmanageable with ligament reconstruction [8].

Corrective surgery is difficult and may not be achievable as a stand-alone operation; moreover a 3D reconstruction to the foot and ankle might be needed in the severe deformity situation. The SMOT (medial open tibia or lateral closing wedge tibia/fibula) has the main role in varus ankle OA correction, along with a calcaneus osteotomy (e.g., Sliding and Dwyer) to address a residual varus hindfoot; a reversed Cotton OT/Dorsiflexion Metatarsal-I-OT to correct the medial foot column; deltoid release or lateral ankle ligament reconstruction to manage deltoid contracture or lateral ankle instability; PL-to-PB-tendon transfer to enhance foot eversion; and posterior tibial tendon elongation or transfer to manage the inversion contracture [8].

### 3.6. Supramalleolar Osteotomy

The aims of supramalleolar osteotomy (SMOT) are to shift the lower-leg/ankle axis to lateral, where there is still good cartilage and to improve intraarticular load distribution by shifting and equalizing the forces and the stresses in the joint and delay the arthritic changes in the tibiotalar joint [15,31]. Multiple factors need to be considered prior to choose your osteotomy. The osteotomy can be performed in one of two ways: medial tibial opening wedge or lateral tibial–fibular closing wedge. The type of SMOT is determined by the deformity’s angle: If the angle is greater than 10 degrees, a lateral closed wedge osteotomy is conceivable; if the angle is less than 10 degrees, a medial open wedge osteotomy is possible [11]. It is wise to mention that, even if a leg length discrepancy up to 10 mm is tolerable according to several studies [32]. Lateral closing wedge SMOT has a greater joint deloading effect and might be more indicated in patients with skin or vascular problems.

## 4. Surgical Technique

### 4.1. Medial Open Wedge Supramalleolar Tibia Osteotomy

General, epidural/spinal, and regional anesthetic are all options for surgery. The patient is positioned supine. To hold the leg in neutral rotation, a slight bump under the ipsilateral hip is frequently required. Then, on the upper leg, a pneumatic tourniquet is applied. Evaluate the imaging before draping to adjust the patient’s position, and apply the stress view to evaluate the ligament integrity. In addition to the preoperative MRI, anterior diagnostic ankle arthroscopy might be performed using standard portals to determine the degree of medial and lateral ankle cartilage loss [5]. The medial approach skin incision is made over the posterior distal tibia, avoiding the saphenous vein and nerve. It is critical to expose the tibia with minimal periosteum peeling in order to complete the osteotomy [32].

With the help of fluoroscopy, a K-wire from medial to the lateral tibial cortex is used to mark the plane of the osteotomy. Two other K-wires orientated anteroposteriorly on the tibia might be of help to control the rotation. Then, the osteotomy is made under Hohmann retractor protection by use of a broad oscillating saw, preserving, if possible, the lateral tibial cortex to act as a fulcrum for the opening wedge and to improve stability [32]. Irrigation is important in order to reduce the heat produced by the saw that may induce damage and slow the postoperative bone healing [32]. Then, the open wedge correction is performed in accordance with the preoperative planning: The mm open wedge distance depicts the amount of angular correction degrees, which were calculated on the standing X-rays preoperatively. The goal is to achieve 2–5° of overcorrection of the MDTA (normal range 93.3 ± 3.2 degrees, value in varus ankle OA < 90°). Preserving the hinge in the lateral cortex is an added value to the osteotomy for the stability and to augment the healing [33]. If there is concern about breaking the lateral cortex while using the lamina spreader to reach the desire correction, a plate on the anterior or lateral tibial cortex gives good control of rotation/translation [32]. Biplanar correction, i.e., adding sagittal correction, is carried out to enhance talar coverage in case of anterior extrusion [33]. A bone autograft or allograft is used to fill the gap, and rigid plate fixation with angular stable screws is used to stabilize the correction. After completion of the tibial osteotomy, the ankle mortise is checked under fluoroscopy to evaluate joint congruency [13]. Joint incongruence can be the result of medial soft tissue contracture, bone formation in the lateral compartment of the ankle, or a too long or malposition fibula. In this case, soft tissue release, cheilectomy, and fibular osteotomy should be carried out until the appropriate position of the talus is obtained. After that, the heel position is evaluated clinically with the goal of achieving 1° to 5° valgus. Deformity that persists should be treated with a calcaneal osteotomy or subtalar arthrodesis [34]. The lateral ankle ligaments and peroneal tendon pathology should also be surgically addressed simultaneously [34]. Additional surgeries are described in Table 3.

### 4.2. Lateral Closing Wedge Supramalleolar Tibia and Fibula Osteotomy

**Figure 2:**
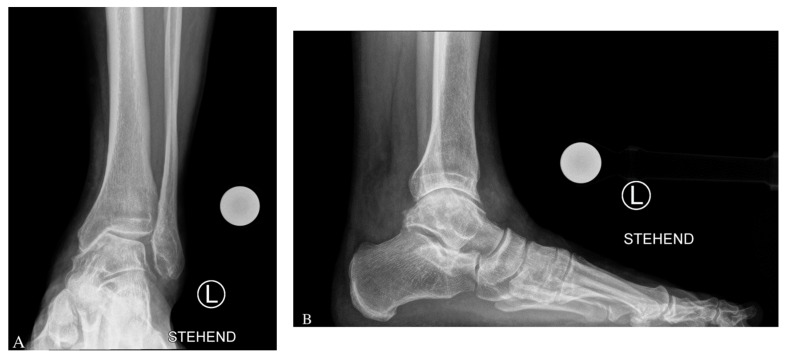

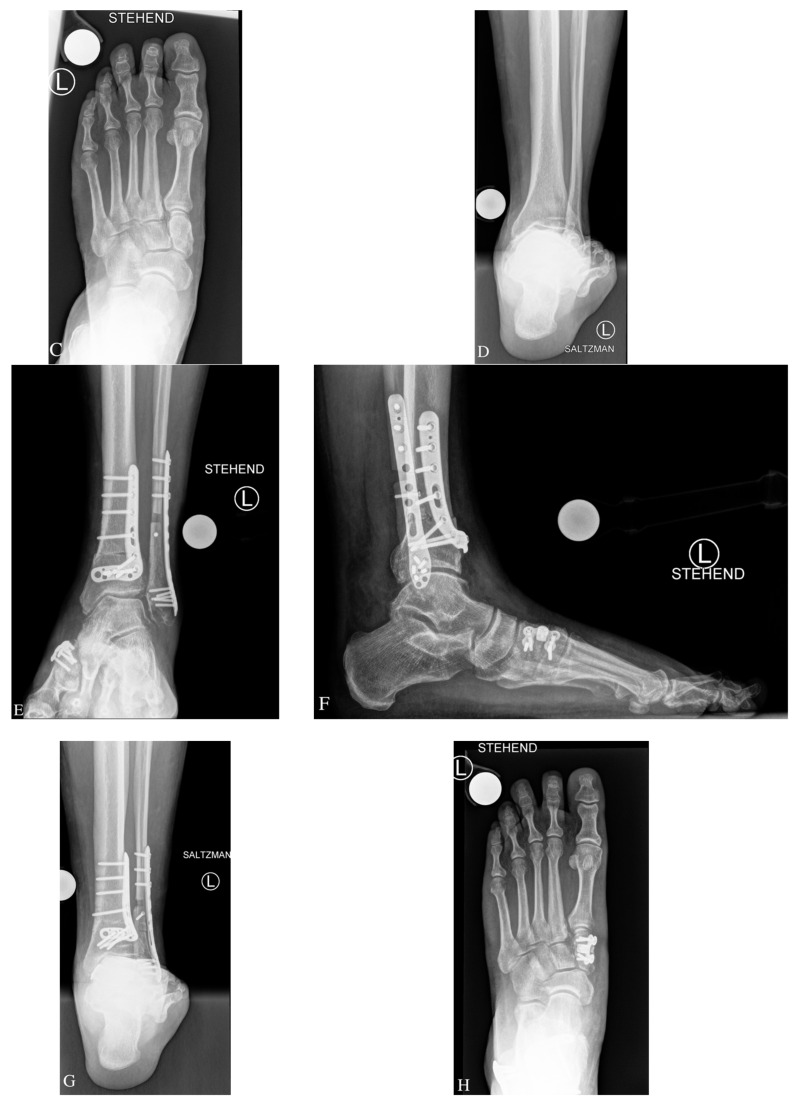
Chronic Painful Posttraumatic Varus Ankle Osteoarthritis (OA) with Medial Degeneration of the Ankle Joint with Pes Planus Foot. In this case, there was preoperative a varus ankle OA with a hindfoot varus and a rare concomitant flatfoot at the midfoot (**A**–**D**). A complex reconstruction was performed (**E**–**H**): Supramalleolar lateral closing wedge osteotomy of the tibia (Anatomical Anterolateral Tibial Plate Aptus, Medartis, Basel, Switzerland), fibular shortening osteotomy (Anatomical Fibular Aptus Plate, Medartis, Basel, Switzerland), anteromedial osteophytes removal/cheilectomy, lateral ankle ligament repair, and Deltoid release. Note that a midfoot Cotton osteotomy (Cotton-Plate with Titanium Wedge, Medartis, Basel, Switzerland) was performed in order to counteract the pre-existing flatfoot deformity.

For lateral closing wedge osteotomy of the fibula and tibia, a lateral approach over the fibula is used [5]. The skin incision starts at the tip of the fibula to proximal over the distal third of the fibula. The fibula, the tibia, and the anterior syndesmosis are then exposed. To preserve ankle joint congruency with a lateral closing wedge osteotomy, shortening of the fibula is needed [5]. The fibula osteotomy is performed by a Z-shaped osteotomy at which the amount of fibula shortening is performed on both ends of the Z. The lateral part of the tibia is then prepared and protected by two Hohmann hooks, dorsal and anterior. Two K-wires are then placed in the tibia above the syndemosis in a converging way in accordance with the amount of correction that was preoperatively planned (MDTA angle) [27]. After that, the osteotomy is performed, and the gap is closed; the correction is stabilized with an angular stable plate. Finally, the position and length of the fibula is adjusted and secured with interfragmentary screws and a fibula plate [27]. See case example in Figure 2.

## 5. Aftertreatment

The aftertreatment after the JPS of varus ankle OA is 15 kg partial weight-bearing with crutches in a walker for 6 weeks, partial increase of load with crutches for further 2 weeks. The patient is treated with physiotherapy from the first day after surgery until 3–4 months postoperative: lymphatic drainage, activation of peroneal muscles, ankle and hindfoot range of motion [5].

## 6. Discussion

Osteoarthritis of the ankle (OA) is a post-traumatic sequela that limits the patient’s activities. The majority of those affected are young and active in sports; therefore, their treatment expectations are higher than those of hip and knee OA patients [8]. Shifting the intraarticular load and stressors from the medial to the lateral ankle (cartilage) by an osteotomy is the best therapeutic option for early-stage varus ankle OA with reasonably well-preserved cartilage [15,35]. The supramalleolar osteotomy (SMOT) is a very effective procedure. It can enhance ankle joint biomechanics, resulting in significant postoperative pain alleviation, improved function, and a slowing of the degenerative process [5,30,31]. Additional procedures are usually required to obtain a fully functional, stable, and plantigrade foot [8].

In the available literature, promising short and midterm results have been documented. Kim et al. [10] assessed 31 ankles with supramalleolar medial opening wedge OT and found considerable pain alleviation (VAS 7.1 + 0.8/3.4 + 1.3) and functional improvement (AOFAS hindfoot score 62.9 + 4.0/83.1 + 7.5) at a mean of 13.2 + 1.4 months after the index surgery. Another study by Pagenstert et al. looked at 35 patients who had supramalleolar osteotomies and found that realignment surgery could delay total ankle replacement or ankle fusion in 91 percent of instances [36]. However, significant pain alleviation should not be expected after an SMOT treatment, due to irreversible pre-existing degenerative changes in the tibiotalar joint and inadequate rectification of the intra-articular deformity. Krähenbühl et al. showed in their prospective study with 99 varus osteoarthritic ankles, a good outcome in the mid- to long-term for the supramalleolar osteotomy (5-year survival rate was 88%) [37]. Age at the time of operation and a preoperative Takakura score of 3b were the two main risk factors for failure after realignment surgery [6]. Recently, the same authors found that patients with a preoperative tilt of the talus in the ankle mortise of 4–10 degrees had a 5-year survival rate of 85% (95% CI, 68–100), while patients with a preoperative tilt of >10 degrees had a survival rate of 65% (95% CI, 46–93; *p* = 0.117) Additionally, Lee et al. considered a preoperative varus tibiotalar tilt of more than 7° to be a potential predictor for a worse postoperative outcome [1].

On the other hand, intra-articular distal tibia osteotomy (plafondplasty) is a possible approach to address varus talar tilt if it persists due to asymmetric joint wear. It can be done simultaneously with medial opening wedge osteotomy as described by Hintermann et al. with a favorable outcome as demonstrated by the mean follow-up of 5.9 years [7]. However, this type of surgery is rarely needed and might not control the ankle OA progression.

In a 2015 study conducted by Haraguchi et al., a hip-to-calcaneus radiograph was used to determine the point of transition of the lower limb mechanical axis at the level of the tibial plafond, expressed as a percentage. Pre and post supramalleolar osteotomy data were then collected, noting that clinically the best AOFAS score values were significantly correlated with a shift in the anatomical axis ≥80%. It was also observed that the point was not relocated far enough to the lateral side in ankles where the preoperative point was more medial than the tibial plafond, therefore the authors suggest, in these cases, to associate additional procedures to lateralize the mechanical axis as much as possible. Moreover, despite the fact that the mean postoperative TAS angle was similar, the positions of the postoperative weight-bearing point varied substantially, suggesting that the TAS angle is not accurate for estimating the tibia’s corrective angle [38].

Gross et al. noted a prognostic indicator and worse outcome once there was a cystic lesion or the lesion was bipolar (kissing lesion in both side), so the surgeon should be cautious once this type of lesion is there [39]. Complications have been stated as rare [40]. The malunion or nonunion rate is up to 22% [40]. Infection and wound healing problems have an incidence reported of up to 22% [40]. The progression of OA after a SMOT was reported to be up to 25% [40]. The revision rate is reported to be 12.5% to 16% [41,42]. The progression of ankle OA and pain can be treated with a total ankle replacement successfully and even easier, or with ankle arthrodesis [40]. In a study from Hintermann et al., 74 patients underwent a SMOT procedure, and <5% required total ankle replacement or arthrodesis [43]. Knupp et al. followed prospectively for 43 months 94 ankles of which 10 ankles (10.6%) were converted to total ankle replacements or fused [44]. Regarding sports and recreation activity, Pagenstert et al. found that SMOT increased sports activity. Improvement in ankle function and pain was correlated with the ability to perform activities symptom-free; however, the frequency of sports had no correlation with patients’ symptoms but showed a higher revision rate. The types of sports activities were mostly low-impact, but high-impact activities, such as jogging and jumping, were also reported [45].

## 7. Conclusions

Several underlying disorders, such as lateral chronic ankle instability, neighboring hindfoot OA, pes cavus, and neuromuscular issues, may lead to varus ankle OA. These conditions should be addressed in addition to deciding on the appropriate intervention due to the cascading events. There are a variety of treatment options available, ranging from conservative to surgical reconstruction. While conservative therapy may be appropriate for patients with early varus ankle OA, surgical reconstruction is required when conservative treatments have failed. The foundation for a good surgical treatment outcome is a thorough diagnostic workup and accurate diagnosis of the unique varus ankle OA. A supramalleolar osteotomy, open or closed wedge depending on the amount of varus, and other surgeries such as lateral ankle ligament restoration, peroneus brevis to longus tendon transfer, plantar fascia release, and reversed Cotton osteotomy are the most prevalent surgical treatments.

## Figures and Tables

**Table 1:** Etiologies of the Varus Ankle Osteoarthritis (OA).

Post-Traumatic	Varus malunion of tibial shaft fractures
	Varus malunion of tibial plafond fractures or malleolar fractures
	Varus malunion of talus and calcaneus fractures
	Avascular necrosis of talus
	Chronic lateral ankle ligament instability
	Postcompartment syndrome
Degenerative	Rheumatoid osteoarthritis
	Varus knee osteoarthritis
	Charcot osteoarthropathy
Neuromuscular	Stroke
	Central and peripheral nerve disorders
	Hereditary motor sensory neuropathy/Charcot–Marie–Tooth disease
	Polio
	Cerebral palsy
	Peroneal brevis muscle insufficiency
	Peroneal tendon ruptures
Congenital	Residual clubfoot (Talipes equinovarus)
	Tarsal coalition
	Excessive tibial external rotation

**Table 2:** Indications, Contraindications, Special risks, and Pitfalls for Joint-Preserving Surgery of Asymmetric Varus Ankle Osteoarthritis (OA).

Indications	Asymmetric medial ankle OA with associated varus deformity and a lateral partially preserved tibiotalar joint
	Osteochondral lesions on the medial talar side of the tibiotalar joint
	Post-traumatic varus deformities after lower leg fractures
	Ankle–hindfoot realignment before or together with total ankle arthroplasty
Contraindications	End-stage OA of the ankle with more than half of the tibiotalar joint surface involved
	Unmanageable ankle–hindfoot instability/neuromuscular imbalance
	Osteomyelitis or infection
	Severe vascular and/or neurologic deficiency
Relative Contraindications	Tobacco use (because of most likely expected high rate of nonunion or delayed union)
	Advanced age (>70 years)
	Patients in poor general health who are unable to accomplish nonweight-bearing rehabilitation after surgery
	Untreated diabetes mellitus (with or without diabetic polyneuropathy)
	Altered bone quality due to medication (e.g., long-term medication with steroids)
	Large cysts
	Osteopenia or osteoporosis
	Untreated rheumatoid osteoarthritis
Special Risks and Pitfalls	Intraoperative injury of neurovascular structures and/or tendons
	Wound healing problems/infections
	Under correction/overcorrection
	Loss of correction due to OA progression
	Delayed union/nonunion
	Hardware removal because of pain/discomfort

**Table 3:** Summary of Associated Deformities and Further Procedures Required in Addition to Supramalleolar Osteotomy (SMOT).

Associated Deformities	Further Procedure Required in Addition to Smot
Osteochondral Lesion of the Medial Ankle (Talus, Tibia, and Plafond)	Autologous Matrix-Induced Chondrogenesis (AMIC)
Ventromedial Bony Ankle Impingement (Osteophytes)	Ventromedial Cheilectomy
Ankle Ligaments: Lateral Chronic Ankle Instability Deltoid Contracture	Anatomical Lateral Ankle Ligament Reconstruction Deltoid Release (at medial malleolus)
Varus Hindfoot with No Subtalar OA	Lateral Sliding Calcaneal Osteotomy/Dwyer Calcaneal Osteotomy
Varus Hindfoot with Subtalar Osteoarthritis	Valgisating Subtalar Arthrodesis
Varus Hindfoot with Hindfoot Osteoarthritis (Subtalar and Talonavicular/Calcaneocuboidal)	Valgisating Triple Arthrodesis
Medial Malleolus Deformity (Erosion, Malposition…)	Medial Malleolus Osteotomy
Tight Gastrocnemius–Soleus Complex	Strayer or Proximal Gastrocnemius Recession
Peroneal Tendon Pathologies Posterior Tibial Tendon Tightness	Primary Repair or PL-to-PB-tendon transfer Posterior Tibial Tendon Elongation
Pes Cavus	Reversed Cotton Osteotomy ± Plantar Fascia Release
Plantar Flexed First Metatarsal	Dorsal Closing Wedge First Metatarsal Osteotomy
Plantar Flexed First Metatarsal with Overdrive of Peroneal Longus Tendon	Dorsal Closing Wedge First Metatarsal Osteotomy and PL-to-PB Tendon Transfer
